# The pseudophosphatase MK-STYX inhibits stress granule assembly independently of Ser149 phosphorylation of G3BP-1

**DOI:** 10.1111/febs.12068

**Published:** 2012-12-07

**Authors:** Justinn E Barr, Michelle R Munyikwa, Elizabeth A Frazier, Shantá D Hinton

**Affiliations:** 1 Department of Biology, Integrated Science Center, College of William and MaryWilliamsburg, VA, USA; 2 Department of Biological Sciences, Hampton UniversityHampton, VA, USA

**Keywords:** G3BP-1, MK-STYX, protein tyrosine phosphatase, pseudophosphatase, stress granules

## Abstract

The pseudophosphatase MK-STYX (mitogen-activated protein kinase phosphoserine/threonine/tyrosine-binding protein) has been implicated in the stress response pathway. The expression of MK-STYX inhibits the assembly of stress granules, which are cytoplasmic storage sites for mRNA that form as a protective mechanism against stressors such as heat shock, UV irradiation and hypoxia. Furthermore, MK-STYX interacts with a key component of stress granules: G3BP-1 (Ras-GTPase activating protein SH3 domain binding protein-1). Because G3BP-1 dephosphorylation at Ser149 induces stress granule assembly, we initially hypothesized that the inhibition of stress granules by MK-STYX was G3BP-1 phosphorylation-dependent. However, in the present study, using MK-STYX constructs and G3BP-1 phosphomimetic or nonphosphorylatable mutants, we show that MK-STYX inhibits stress granule formation independently of G3BP-1 phosphorylation at Ser149. The introduction of point mutations at the ‘active site’ of MK-STYX that convert serine and phenylalanine to histidine and cysteine, respectively, is sufficient to generate an active enzyme. In separate experiments, we show that this active mutant, MK-STYX_active_, has opposite effects to wild-type MK-STYK. Not only does MK-STYX_active_ induce stress granules, but also it has the capacity to dephosphorylate G3BP-1. Taken together, these results provide evidence that the pseudophosphatase MK-STYX plays a key role in the cellular response to stress.

## Introduction

Protein phosphorylation signalling cascades regulate many diverse cellular responses, including the stress response. Kinases and phosphatases control the activity of target proteins by coordinating a fine balance between phosphorylation and dephosphorylation. Disruption of this balance leads to diseases such as cancer, diabetes, chronic inflammation and Alzheimer's. In a newly recognized level of complexity, phosphorylation cascades are further modulated by a variety of pseudokinases and pseudophosphatases 1–5, which lack catalytic activity but have homology to kinases and phosphatases, respectively. An intriguing advance in the field was the discovery of naturally existing ‘substrate-trapping mutants’. In these mutants, an inactive version of the catalytic domain converts extremely active protein tyrosine phosphatases (PTPs) into substrate traps 6. Over the past 15 years, the development of synthetic ‘substrate-trapping’ mutants has helped identify many PTP substrates and advanced our understanding of the physiological roles of PTPs. However, it remains equally important to characterize the function of naturally-occurring pseudophosphatases, which also have the ability to bind phosphorylated residues 4,5.

Many pseudophosphatases have been identified 7, yet the function of most of these catalytically-dead members of the PTP family remains elusive. Other members of the PTP family have an active site signature motif 3, in which a nucleophilic cysteine is essential for catalytic activity. However, pseudophosphatases lack critical residues within their active site, preventing enzymatic activity 5. Reports have shown that a single point mutation that converts a substituted amino acid in the active site back to a cysteine restores enzymatic activity 1,4. In addition, the classical PTP fold is preserved in pseudophosphatases, thus maintaining their ability to bind phosphorylated target proteins 6,8,9. Initially, it was proposed that these proteins would serve as naturally-occurring substrate-trapping mutants that block the function of endogenous phosphatases 7. However, the prototypic pseudophosphatase STYX (phosphoserine/threonine/tyrosine-binding protein), which has a glycine residue instead of cysteine in its active site 4,5, interacts with the RNA-binding protein CRHSP24 (calcium-responsive heat-stable protein with a molecular mass of 24 kDa) 10. Furthermore, the interaction of STYX with CRHSP24 is essential for spermatogenesis in mice 10. Besides STYX, a number of pseudophosphatases may regulate signalling cascades 7,11,12 in ways other than by merely preventing dephosphorylation of target phosphate groups. For example, some pseudophosphatases engage in direct protein–protein interactions rather than protein–phosphate binding interactions with their target proteins 7. To date, the myotubularin (MTM) family is the most prevalent group of pseudophosphatases involved in such interactions. For example, the pseudophosphatase MTM-2 forms a complex with its active homologue MTM-13, and MTM-2 also regulates the function and subcellular localization of the active phosphatase 13. Furthermore, a mutation in either protein in a MTM pseudophosphatase–phosphatase complex such as MTM-13 or MTM-2 results in Charcot–Marie–Tooth disease, which is characterized by abnormal nerve myelination 14. Pseudophosphatases not only interact with phosphatases, but also form complexes with kinases to regulate their activity 7,12,15. In particular, the *Caenorhabditis elegans* pseudophosphatases EGG-4 and EGG-5 inhibit the activity of the dual-specificity tyrosine-regulated kinase minibrain kinase homologue-2 (MBK-2) by binding to its activation loop, preventing substrate phosphorylation 7,15.

In the present study, we focus on the pseudophosphatase MK-STYX, a member of the mitogen-activated protein kinase (MAPK) phosphatase class 1,3,5. Whereas catalytically active phosphatases include the signature motif **HC**(X_5_)**R**, the corresponding MK-STYX sequence (I**FS**TQGIS**R**S) lacks both the critical histidine and cysteine residues 1,3,16. As in other cases, mutations that restore this signature motif, I**HC**TQGIS**R**S, create an active phosphatase 1. In its native, catalytically-inactive form, wild-type MK-STYX functions in a number of important cellular pathways. Conversely, alterations in MK-STYX expression are associated with tumorigenesis; for example, MK-STYX is highly expressed in Ewing's sarcoma cells 17.

In earlier studies concerning the cellular functions of MK-STYX, we discovered that MK-STYX binds directly to a protein that regulates both the Ras signalling pathway and stress granule assembly 1. This regulator, G3BP-1 (Ras-GTPase activating protein SH3 domain binding protein-1), is ubiquitously expressed and contains several distinct domains that enable it to interact with multiple protein and RNA-binding partners 18,19. Furthermore, G3BP-1 is also widely accepted as a marker and nucleator of stress granules 20,21. In particular, studies of G3BP-1 and/or various G3BP-1 mutants have revealed important insights into the dynamic assembly of stress granules 22–25. Although G3BP-1 has multiple phosphorylation sites, only Ser149 is considered to be significant for stress granule assembly 23. Specifically, mammalian cells expressing either G3BP-1 or a nonphosphorylatable G3BP-1 mutant (S149A) induce stress granule assembly, whereas the phosphomimetic mutant (S149E) does not induce stress granule assembly 23.

The present study focuses on understanding how MK-STYX inhibits stress granule formation. Initially, we hypothesized that MK-STYX inhibited stress granule formation by locking G3BP-1 in its phosphorylated state. However, in the present study, we show that, in heat-shocked HeLa cells, the expression of MK-STYX significantly inhibits stress granule formation in the presence of either phosphomimetic (S149E) or nonphosphorylatable (S149A) G3BP-1 mutants. These data indicate that MK-STYX inhibits stress granule formation independently of the phosphorylation state of G3BP-1 at Ser149, and thus suggest that MK-STYX acts at another point in the signalling pathway. Nevertheless, an interaction between MK-STYX and G3BP-1 is supported both by our immunoprecipitation studies 1, as well as by the observation that the catalytically-active mutant of MK-STYX was able to dephosphorylate G3BP-1 specifically at Ser149 and induce endogenous stress granules. Furthermore, the presence of the active mutant induced the formation of stress granules by the phosphomimetic G3BP-1 mutant (S149E), as well as larger granules in the presence of S149A-G3BP. Taken together, these data provide evidence for a role of MK-STYX as a regulator in the stress response pathway.

## Results

### MK-STYX inhibits G3BP-1-induced stress granule formation

**Fig 1:**
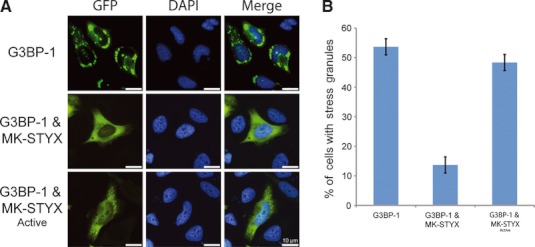
MK-STYX inhibits G3BP-1 induced stress granules. (A) Representative examples of the subcellular distribution patterns of G3BP-1. HeLa cells cotransfected with expression vectors for G3BP-1 and wild-type MK-STYX showed fewer cells with stress granules. Overexpression of G3BP-1 and MK-STYX active mutant resulted in intermediate stress granule assembly. Merged images show the location of GFP-tagged G3BP-1 (green) relative to DAPI-stained nuclei (blue). Scale bar = 10 μm. (B) HeLa cells were cotransfected with expression vectors for G3BP1-GFP and either MK-STYX or MK-STYX active mutant. Cells were analyzed 24 h post-transfection for G3BP-induced stress granule assembly by fluorescence microscopy, after staining with DAPI to reveal the nuclei. Cells were scored for the presence or absence of stress granules. Three replicate experiments were performed (*n* = 100); error bars indicate the SEM.

Stress granule assembly can be induced by a variety of stressors, including the overexpression of G3BP-1 and the dephosphorylation of G3BP-1 at Ser149 23. Conversely, stress granule assembly can be blocked by the overexpression of MK-STYX 1. To test whether MK-STYX could inhibit stress granule formation induced by phosphomimetic and nonphosphorylatable mutants of G3BP-1, we first needed to establish a baseline for analyzing changes in the pattern of stress granule assembly for wild-type G3BP-1. Accordingly, we expressed wild-type green fluorescent protein (GFP)-tagged G3BP-1 either alone, or together with wild-type FLAG-tagged MK-STYX or the catalytically active mutant of MK-STYX (MK-STYX_active_) (Fig. 1). As anticipated from our previous studies 1, G3BP-1 overexpression induced the formation of large, perinuclear stress granules, whereas stress granule formation was markedly inhibited by the co-expression of MK-STYX. By contrast, the co-expression of MK-STYX_active_ induced the formation of intermediate granules (smaller aggregates dispersed throughout the cytoplasm). Because we had previously shown that MK-STYX does not suppress the expression of G3BP-1 1, this inhibition of stress granule formation is not the result of a lack of G3BP-1 expression.

### Inhibition of stress granule assembly by MK-STYX is independent of phosphorylation of G3BP-1 at Ser149

**Fig 2:**
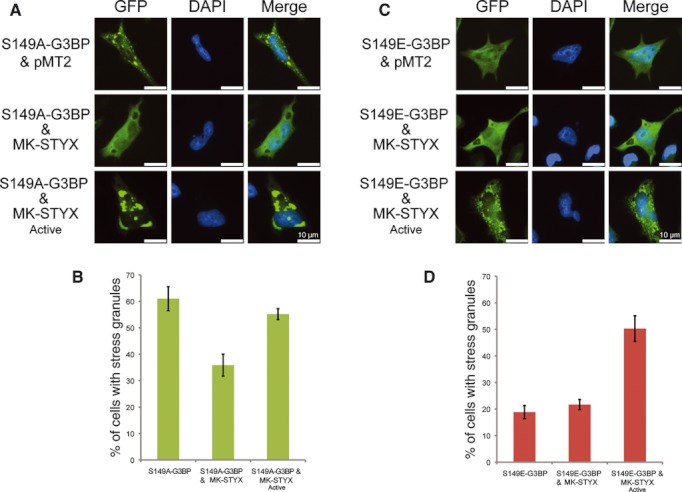
Phosphorylation-independent inhibition of stress granule assembly by MK-STYX. (A, C) Representative examples of the subcellular distribution patterns of GFP-tagged G3BP-149 mutants. HeLa cells cotransfected with expression vectors for S149A-G3BP and wild-type MK-STYX showed fewer cells with stress granules. The overexpression of S149A-G3BP alone resulted in stress granule assembly. Cells cotransfected with S149A-G3BP and the active MK-STYX mutant accumulated larger perinuclear granules. Cells expressing S149E-G3BP mutant alone or in the presence of MK-STYX did not accumulate stress granules, whereas cells expressing S149E-G3BP and the MK-STYX active mutant accumulated smaller granules that were more dispersed throughout the cytoplasm. Merged images show the location of GFP-tagged G3BP-1 mutants (green) relative to DAPI-stained nuclei (blue). Scale bar = 10 μm. (B, D) HeLa cells were cotransfected with expression vectors for GFP-tagged G3BP-149 mutants, and either MK-STYX or MK-STYX active mutant. Cells were analyzed 24 h post-transfection for G3BP-induced stress granule assembly by fluorescence microscopy, after staining DAPI to reveal the nuclei. Cells were scored for the presence or absence of stress granules. Three replicate experiments were performed (*n* = 100); error bars indicate the SEM.

Dephosphorylation of G3BP-1 at Ser149 has been shown to induce stress granule assembly 23. To determine whether the ability of MK-STYX to inhibit stress granule assembly requires phosphorylation of G3BP-1 at Ser149, we co-expressed either the wild-type or active mutant form of MK-STYX with GFP-tagged versions of either S149A-G3BP or S149E-G3BP (Fig. 2). Consistent with earlier studies 23, the expression of nonphosphorylatable S149A-G3BP alone resulted in stress granule assembly in ≥ 60% of the transfected cells (Fig. 2A,B), whereas the expression of the phosphomimetic S149E-G3BP alone was associated with low levels of stress granule assembly (< 20%) (Fig. 2D).

To determine how the pseudophosphatase MK-STYX might alter these levels, we co-expressed the S149A-G3BP mutant with MK-STYX. Based on our initial model, we predicted that MK-STYX would inhibit stress granule formation by shielding the phosphate group at Ser149 of G3BP-1 from phosphatase activity; hence, the expression of MK-STYX was not expected to alter stress granule formation. However, when we measured stress granule formation in cells co-expressing MK-STYX and S149A-G3BP, MK-STYX was found to suppress stress granule assembly (Fig. 2A,B). This unexpected result indicates that the ability of MK-STYX to inhibit stress granule assembly does not solely depend on the phosphorylation state of G3BP-1 at residue 149.

Given the inhibitory effect of MK-STYX, we next tested whether the expression of the phosphatase-active mutant of MK-STYX (MK-STYX_active_) would enhance stress granule formation in cells co-expressing either the nonphosphorylatable or phosphomimetic G3BP-1 mutants. In the presence of MK-STYX_active_, not only did cells expressing the nonphosphorylatable S149A-G3BP mutant form stress granules (Fig. 2B), but also the stress granules that formed were notably larger (Fig. 2A). Furthermore, co-expression of MK-STYX_active_ with the phosphomimetic S149E-G3BP mutant resulted in more cells with stress granules overall and stress granules that were intermediate in size (Fig. 2C), consistent with studies of wild-type G3BP-1 (Fig. 1A,B) 1. Overall, ∼ 53% of cells expressing the phosphomimetic S149E-G3BP and MK-STYX_active_ formed stress granules (Fig. 2C,D). The additive effect of larger stress granules forming in the presence of the active mutant of MK-STYX may suggest an interaction between MK-STYX and microtubules. Previous studies have suggested that larger aggregates form when microtubules transport smaller aggregates to a localized area 26–28. In our studies, the induction of larger granules in cells co-expressing the active mutant and nonphosphorylatable S149A-G3BP suggests that the active mutant may initiate and/or facilitate the transport of smaller aggregates to a localized area.

**Fig 3:**
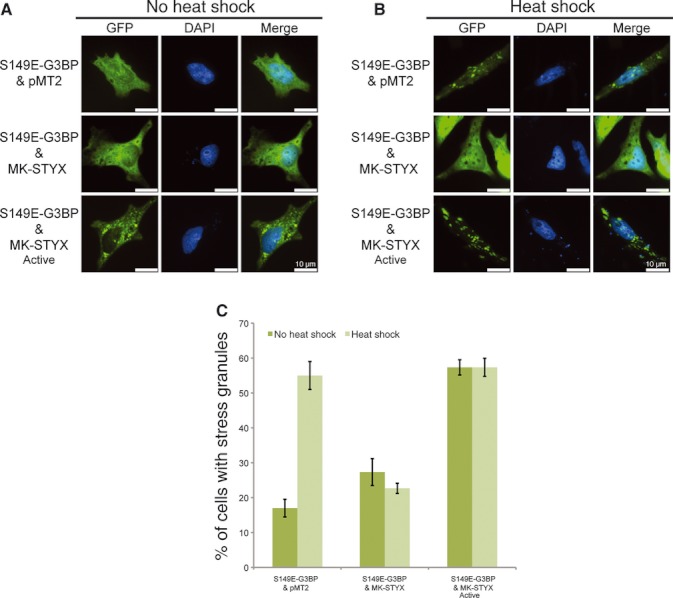
Induction of S149E-G3BP stress granule assembly by the MK-STYX active mutant. Representative examples of the subcellular distribution of GFP-tagged S149E-G3BP without heat shock (A) or after heat shock at 41 °C (B), in the presence of pMT2 (empty vector), MK-STYX or the active mutant as indicated. Cells were fixed, stained with DAPI and analyzed 24 h post-transfection for heat shock-induced stress granule assembly by fluorescence microscopy. Merged images show the localization of S149E-G3BP (green) relative to the DAPI-stained nucleus (blue). Scale bar = 10 μm. (C) Cells were scored for the presence or absence of stress granules. Three replicate experiments were performed (*n* = 100 cells for each experiment); error bars indicate the SEM.

Transient overexpression of the stress granule-associated protein G3BP-1 is a commonly used approach for investigating stress granule assembly because it results in the appearance of stress granules in the absence of external stressors 29. However, in some cases, when mutant forms of G3BP-1 are being investigated, external stressors must be used to induce stress granule formation 29**.** Thus, to investigate the effects of MK-STYX on the G3BP-1 phosphomimetic mutant, we heat-shocked cells expressing the phosphomimetic S149E-G3BP mutant together with either MK-STYX or MK-STYX_active_ (Fig. 3). As expected, we observed fewer cells with stress granules under both the nonheat-shocked conditions (Fig. 3A) and in cells expressing MK-STYX under heat-shocked conditions (Fig. 3C). Once again, the active mutant induced intermediate stress granule formation in unstressed cells that were also expressing the phosphomimetic S149E-G3BP-1 mutant (Fig. 3A). Approximately 55% of heat shock-stimulated cells expressing S149E-G3BP formed stress granules (Fig. 3B,C). These data are consistent with a previous study demonstrating that, in the presence of S149E-G3BP, arsenite-stressed Cos cells formed stress granules 23. Although heat shock induced the formation of stress granules in cells expressing S149E-G3BP, co-expression of MK-STYX prevented their formation (Fig. 3B,C). Thus, the interaction between the G3BP-1 mutants and MK-STYX may elicit a conformational change in G3BP-1 that prevents stress granule formation. Taken together, these results imply that the mechanism by which the pseudophosphatase MK-STYX prevents stress granule formation involves complexities beyond protecting G3BP-1 at phospho-S149.

### Expression of the active mutant of MK-STYX induces stress granule formation

**Fig 4:**
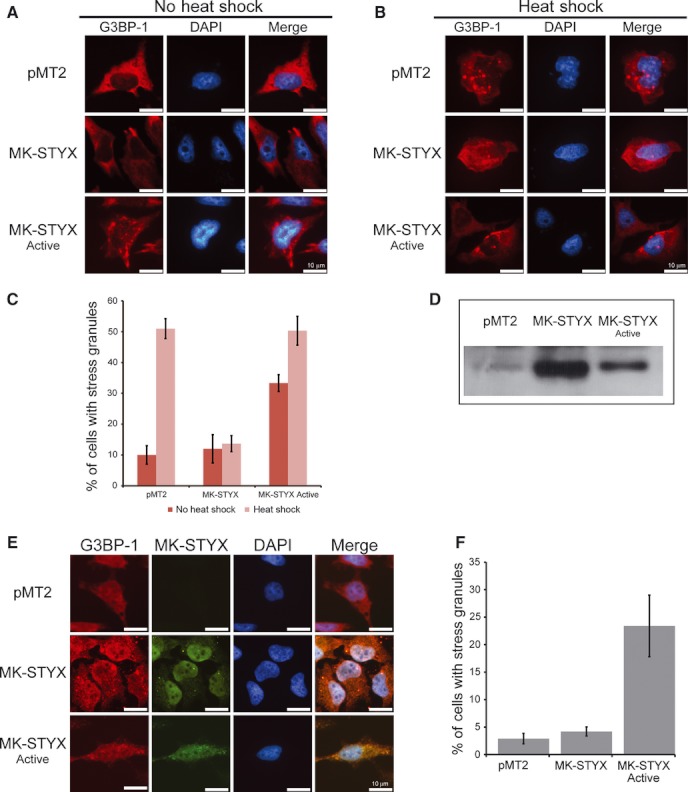
Inhibition of heat shock-induced stress granule formation by MK-STYX Representative examples of the subcellular distribution of endogenous G3BP-1 without heat shock (A) or after heat shock at 41 °C (B), in the presence of pMT2 (empty vector), MK-STYX or the active mutant as indicated. Cells transfected with MK-STYX did not form heat shock-induced granules, whereas the active mutant induced granules in the presence or absence of heat shock. Cells were fixed, stained with anti-G3BP and Cy3-conjugated goat anti-mouse sera and DAPI, and analyzed 24 h post-transfection for heat shock-induced stress granule assembly by fluorescence microscopy. Merged images show the localization of endogenous G3BP-1 (red) relative to DAPI-stained nuclei (blue). Scale bar = 10 μm. (C) Cells were scored for the presence or absence of stress granules. Three replicate experiments were performed (*n* = 100 cells for each experiment); error bars indicate the SEM. (D) Blots were probed with anti-FLAG to visualize the presence of MK-STYX or active mutant. (E) Representative examples of the subcellular distribution of endogenous G3BP-1 and MK-STYX or MK-STYX_active_. Cells were fixed, stained with anti-G3BP and Cy3-conjugated goat anti-mouse sera, FLAG conjugated to FITC (anti-FLAG-FITC) for the detection of FLAG-tagged MK-STYX or MK-STYX_active_ and DAPI. (F) Cells overexpressing MK-STYX or MK-STYX_active_ (green) were scored for the presence or absence of stress granules. Merged images show the localization of endogenous G3BP-1 (red) relative to MK-STYX (green), MK-STYX_active_ (green) or DAPI-stained nuclei (blue). Scale bar = 10 μm.

Because MK-STYX_active_ induced stress granules in the presence of the G3BP-1 phosphomimetic mutant, we aimed to determine whether expression of the active mutant alone could induce endogenous stress granules. We compared the effects of heat shock on stress granule formation in cells transfected with either empty vector as a control, or MK-STYX or MK-STYX_active_ expression plasmids. Endogenous G3BP-1, a nucleator of stress granules 23, was used as the marker for stress granule formation. The expression of endogenous G3BP-1 was visualized by indirect immunofluorescence with antibodies specific for G3BP-1 (Fig. 4). Heat shock induced stress granule formation in ~ 50% of the control cells. As expected, the expression of MK-STYX significantly suppressed stress granule formation; only ∼ 15% of cells ectopically expressing MK-STYX formed granules (Fig. 4B,C). By contrast, MK-STYX_active_ promoted the formation of stress granules in both nonstimulated and heat-shocked cells (Fig. 4A–C). Expression of both FLAG-tagged MK-STYX and the active mutant was detected in cell lysates with anti-FLAG sera (Fig. 4D).

To test whether MK-STYX co-localizes with G3BP-1, we double-stained cells by indirect immunofluorescence using anti-G3BP-1 and anti-FLAG sera. We compared cells expressing MK-STYX with cells expressing MK-STYX_active_ and scored only the cells expressing MK-STYX or MK-STYX_active_ for stress granule formation (Fig. 4E). Approximately 23% of cells expressing the active mutant assembled stress granules, whereas MK-STYX was comparable to the control (Fig. 4F). Furthermore, MK-STYX_active_ co-localized with endogenous G3BP-1 to a greater extent than MK-STYX (Fig. 4E), suggesting that the active mutant may dephosphorylate MK-STYX.

### MK-STYX_active_ dephosphorylates G3BP-1 at Ser149

**Fig 5:**
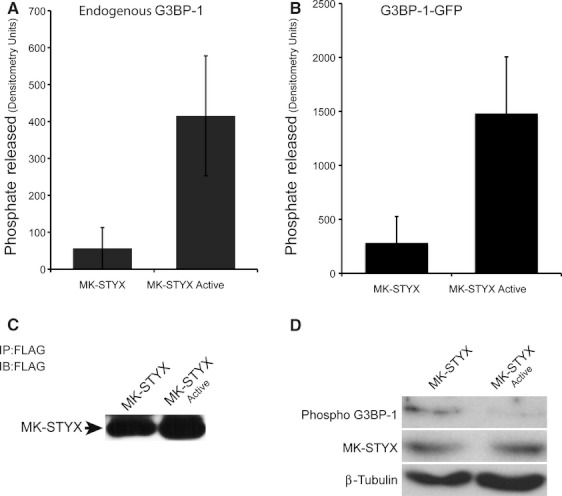
MK-STYX active mutant dephosphorylates G3BP-1. HeLa cells or HeLa cells expressing G3BP-1-GFP were labelled with [γ-^32^P]ATP, lysed and immunoprecipitated with anti-G3BP to obtain ^32^P-G3BP as substrate for a phosphatase liquid assay. Wild-type pMT2-FLAG-MK-STYX-FLAG and active mutant MK-STYX were expressed in HeLa cells, immunoprecipitated with anti-FLAG and assayed for phosphatase activity against (A) ^32^P-G3BP or (B) ^32^P-G3BP-GFP immunoprecipitates as substrate. The active mutant dephosphorylated endogenous G3BP-1, as well as G3BP-1-GFP. Three replicate experiments were performed. Error bars indicate the SEM. (C) An aliquot of the anti-FLAG immunoprecipitate (IP) used in the assays was resolved by SDS/PAGE and analyzed. Blots (IB) were probed with anti-FLAG to visualize immunoprecipitated MK-STYX, confirming the presence of wild-type and active mutant MK-STYX in the phosphatase assays. (D) HeLa cells expressing MK-STYX or MK-STYX_active_ were lysed and resolved by SDS/PAGE and analyzed. Blots were probed with anti-phosphoG3BP149 to determine the phosphorylation status of G3BP-1 at Ser149, anti-FLAG to confirm the presence of MK-STYX and β-tubulin as a loading control.

The observation that MK-STYX_active_ induces stress granules and colocalizes with endogenous G3BP-1 suggested that this catalytically active form might directly dephosphorylate G3BP-1. To specifically test this point, ^32^P-labelled endogenous G3BP-1 and ectopically expressed GFP-tagged G3BP-1 were immunoprecipitated and used as substrates (Fig. 5). Immunoprecipitated MK-STYX_active_ dephosphorylated both endogenous G3BP-1 (Fig. 5A) and ectopically expressed GFP-tagged G3BP-1 (Fig. 5B). By contrast, immunoprecipitates of wild-type MK-STYX did not dephosphorylate either endogenous G3BP-1 or G3BP-GFP. Both MK-STYX and the active mutant were detected in immunoprecipitates with anti-FLAG sera (Fig. 5C), indicating that differences in phosphatase activity were not a result of altered protein levels.

To determine whether MK-STYX_active_ specifically dephosphorylates G3BP-1 at site 149, western blots were performed with an antibody specific for G3BP-1 phosphorylation at Ser149 [anti-phospho-G3BP (pSer^149^)]. In the presence of MK-STYX_active_, phosphoG3BP-1 at site 149 was not detectable (Fig. 5D), suggesting that the active mutant dephosphorylates MK-STYX. By contrast, phosphoG3BP-1 at site 149 was detectable in the presence of wild-type MK-STYX (Fig. 5D).

Taken together, our results demonstrate that wild-type MK-STYX and its active mutant elicit opposite effects on stress granule formation. Furthermore, including MK-STYX_active_ in these studies for comparison not only supports, but also strengthens the idea that pseudophosphatases have specific and important roles in signalling cascades.

## Discussion

**Fig 6:**
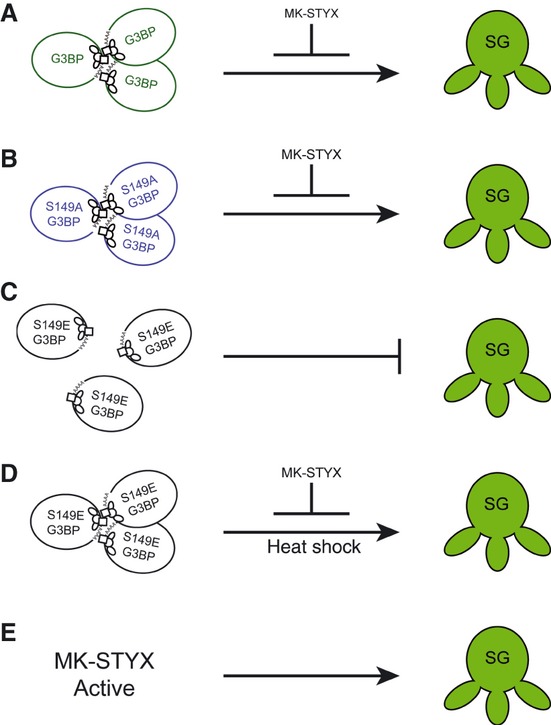
A model of the effects of MK-STYX or MK-STYX_active_ on G3BP-1-induced stress granules. (A) Overexpression of G3BP-1 induces stress granules, which are inhibited by MK-STYX. (B) MK-STYX inhibits stress granule formation by the G3BP-1 nonphosphorylatable mutant S149A-G3BP-1. (C) The phosphomimetic mutant S149E-G3BP-1 does not aggregate to form stress granules. (D) Heat shock induces S149E-G3BP-1 stress granules, although their formation is blocked by MK-STYX. (E) MK-STYX_active_ induces stress granules.

The ability of the pseudophosphatase MK-STYX to inhibit stress granule formation was originally assumed to depend solely on its interaction with phosphorylated G3BP-1. However, the data reported in the present study are inconsistent with this model. Instead, our studies with MK-STYX constructs and G3BP-1 mutants indicate that MK-STYX may act beyond the G3BP-1 signalling pathway to regulate stress granule formation. The results obtained dispel the notion that MK-STYX inhibits stress granules by simply protecting phosphorylation of G3BP-1 at Ser149. Instead, the data suggest a new model that takes into account this more complicated interaction between the pseudophosphatase and G3BP-1 (Fig. 6). In summary, the pseudophosphatase MK-STYX inhibits G3BP-1-induced stress granule formation (Fig. 6A,B,D), whereas the active mutant, MK-STYX_active_, induces stress granule formation and, in some cases, induces larger stress granule aggregates (Fig. 6E). Furthermore, the nonphosphorylatable mutant S149E-G3BP-1 is unable to induce stress granules (Fig. 6C), except in the presence of MK-STYX_active_, pointing to the importance of a conformational change in G3BP-1 for aggregation and stress granule formation. Finally, these studies demonstrate the importance of comparing a naturally-occurring pseudophosphatase with its mutated catalytically-active form. In this case, the comparison clearly demonstrates the distinct interaction of the pseudophosphatase MK-STYX with G3BP-1; MK-STYX inhibits stress granule assembly.

Dephosphorylation of G3BP-1 at site 149 is essential for stress granule assembly 23; however, the mechanism is not well understood. In the present study, the co-expression of MK-STYX and a G3BP-1 nonphosphorylatable mutant (S149A-G3BP) revealed that MK-STYX is capable of inhibiting stress granule assembly independently of G3BP-1 phosphorylation status at Ser149. Furthermore, the active mutant of MK-STYX induced stress granules. Although the mode of action of MK-STYX remains to be determined, the findings reported in the present study enhance our understanding of its interaction with G3BP-1, and provide new insight into the role of MK-STYX in the stress granule life cycle. In the broader perspective, our findings point to the possibility that pseudophosphatases are not simply ‘protectors’ of phosphorylated proteins but, instead, are important regulators of signalling cascades, including the stress response pathway.

The finding that wild-type MK-STYX and MK-STYX_active_ elicit opposite effects on stress granule formation is insightful to our understanding of the molecular mechanism of the pseudophosphatase MK-STYX. Furthermore, our immunoblotting results suggest that MK-STYX_active_ dephosphorylates G3BP-1 at Ser149, indicating the possibility that wild-type MK-STYX interacts with G3BP-1 at this site. However, our results demonstrate that phosphorylation of S149 is not required for MK-STYX to inhibit G3BP-1-induced stress granule formation. This raises the question of how MK-STYX might inhibit stress granule formation. The answer may lie within other key characteristics of G3BP-1.

G3BP-1 originally was purified in a complex with RasGAP (Ras GTPase activating protein) 30, which is essential for Ras signalling. The G3BP–GAP complex only forms in proliferating cells, suggesting that Ras must be in its activated conformation for G3BP and RasGAP interaction 30. Furthermore, the phosphorylation pattern of G3BP-1 differs in quiescent and proliferating cells; it is highly phosphorylated on multiple serine residues in quiescent cells, although proliferating cells lack phosphorylation at Ser149 18. The absence of a phosphate group on Ser149 in proliferating cells was shown to be dependent on RasGAP, as well as to influence the subcellular distribution of G3BP-1 18**.** The normally cytosolic protein translocates to the nucleus when phosphorylated at Ser149 22. The fact that MK-STYX is a member of the MAPK phosphatase class of PTPs, which regulates downstream MAPKS effectors of Ras 31, provides another link for MK-STYX as a stress regulator. In addition to MK-STYX acting as a stress-linked regulator, a recent study suggested that MK-STYX also acts as a gatekeeper to mitochondrial cell death in the apoptotic pathway 32. Thus, MK-STYX may play a pivotal role in a number of cell signalling pathways.

Our results demonstrate that the phosphorylation of G3BP-1 has different effects on MK-STYX and MK-STYX_active_. Furthermore, the active mutant induces the assembly of intermediate-sized granules in cells expressing phosphomimetic S149E-G3BP-1. In the absence of MK-STYX_active_, cells expressing S149E-G3BP-1 do not exhibit elevated levels of stress granule formation. This result suggests that the active mutant causes a conformational change, independent of dephosphorylation, that induces stress granule assembly. The N-terminus of G3BP-1 is characterized by the presence of a nuclear transport factor 2 (NTF2)-like domain, and a segment rich in acidic acids that has a key role in protein–protein interactions 23. Previous studies showed that the Ser149 residue essential for stress granule formation lies 20 amino acids upstream of this NTF2 domain 23. Furthermore, the NTF2 domain promotes G3BP-1 aggregation, which is prevented when G3BP-1 is phosphorylated at Ser149 23. Perhaps the interaction of the wild-type pseudophosphatase MK-STYX with G3BP-1 causes a conformational change in its binding partners that prevents the aggregation of the NTF2 domains of G3BP-1. This paradigm has been reported with the pseudophosphatases EGG-4 and EGG-5. Their interaction with the kinase MBK-2 prevents accessibility to the kinase activation loop 15. Furthermore, EGG-4 and EGG-5 are able to bind the MBK-2 YTY motif regardless of phosphorylation 15. We report similar data in the present study with the pseudophosphatase MK-STYX, which inhibits stress granule formation in the presence of G3BP-1 nonphosphorylated or phosphorylatable mutants.

In conclusion, the data obtained in the present study enhance our understanding of MK-STYX and its effects on stress granule dynamics, offering insights into G3BP-1-induced stress granule formation. Our data provide evidence that MK-STYX prevents stress granule accumulation independently of G3BP-1 phosphorylation at site 149. It will be of interest to investigate the dynamics between MK-STYX and G3BP-1 that result in the inhibition of stress granules. For example, is the role of MK-STYX in stress granule dynamics via polysome accumulation or stress granule disassembly? The precise mechanism of the pseudophosphatase MK-STYX still remains to be defined. However, our results dispel the notion that pseudophosphatases serve exclusively as ‘protectors’ that block protein tyrosine phosphatases. Instead, they support a model in which pseudophosphatases have very distinctive roles in regulating phosphorylation cascades. Taken together, our findings point toward a pivotal role of the pseudophosphastase MK-STYX as a *bona fide* modulator in the stress response signalling pathway.

## Experimental procedures

### Plasmid constructs

pMT2-FLAG-MK-STYX-FLAG and pMT2-FLAG-MK-STYX_active_-FLAG were generated as described previously by Hinton *et al*. 2010. The integrity of all constructs derived from PCR was confirmed by DNA sequencing. The G3BP-1-GFP, S149A-G3BP-GFP and S149E-G3BP-GFP constructs were kindly provided by Jamai Tazi (Institut de Génétique Moléculaire, Montpellier, France).

### Cell culture and transient transfection

HeLa cells were maintained at 37 °C in 5% CO_2_ in MEM (Invitrogen, Carlsbad, CA, USA) supplemented with 10% fetal bovine serum. Transfections were performed using Lipofectamine 2000 (Invitrogen). When heat shock experiments were required, cells were maintained at 41 °C for 1 h followed by immediate fixation or lysing for either immunoprecipitation or immunoblotting.

### Transient transfection and cell imaging

For immunofluorescence assays, HeLa cells were grown to 80–90% confluence and 2 × 10^5^ cells were plated onto coverslips in six-well dishes (Nunc, Rochester, NY, USA). Twenty-four hours post-plating, cells at 40–60% confluence were transfected with 2 μg of G3BP-1, S149A-G3BP-GFP, S149E-G3BP-GFP, pMT2, or wild-type or mutant FLAG-tagged MK-STYX expression plasmid DNA and 4 μL of Lipofectamine 2000 per well, in accordance with the manufacturer's instructions. The medium was replaced 5 h after transfection. Twenty-four hours post-transfection, cells were washed with PBS and fixed with 3.7% formaldehyde. The coverslips were mounted to a slide using GelMount containing 4′,6-diamidino-2′-phenylinodole dihydrochloride (DAPI; Sigma, St Louis, MO, USA) (0.5 mg·mL^−1^).

For experiments examining the effect of MK-STYX and the active mutant on stress-induced stress granule assembly, cells were transfected with S149A-G3BP-GFP or S149E-G3BP-GFP and either pMT2, wild-type or mutant FLAG-tagged MK-STYX expression plasmid DNA. The medium was replaced 5 h after transfection and, at 23 h post-transfection, cells were stressed at 41 °C for 1 h, and then processed as above. For localization studies, cells were transfected with pMT2, MK-STYX or MK-STYX_active_. To visualize stress granule assembly with cells expressing MK-STYX or MK-STYX_active_, endogenous G3BP-1 was used as the marker and visualized with anti-G3BP (dilution 1 : 100) and Cy3 (indocarbocyanine)-conjugated goat anti-mouse (dilution 1 : 200; Zymed Laboratories Inc., San Francisco, CA, USA) sera. To visualize the localization of MK-STYX or MK-STYX_active_, anti-FLAG-fluorescein isothiocyanate (FITC) (dilution 1 : 100; Sigma) sera was used.

Counting and image collection were performed on an Olympus BX60 microscope with U-MNU filter cube for DAPI and Omega Optical XF100-2 for GFP and an Olympus × 40 UPlanFL × 40/0.75 objective (Olympus, Tokyo, Japan). A Cooke SensiCam camera and iplab software (Becton-Dickinson Biosciences, Franklin Lakes, NJ, USA) were used for image acquisition and primary image processing. imagej (NIH, Bethesda, MD, USA), adobe photoshop and adobe illustrator (Adobe Systems, San Jose, CA, USA) were used for secondary image processing.

Approximately 100 cells were scored per treatment. Samples were scored blind with respect to treatment and were scored independently by two different individuals. Cells were scored into two categories: no stress granules and stress granules.

### Metabolic labelling

Forty-eight hours after post-transfection with G3BP-1-GFP, HeLa cells were incubated for 30 min in DMEM minus phosphate (Invitrogen), supplemented with 10% dialyzed fetal bovine serum and 1 : 100 l-glutamine. Subsequently, cells were incubated for 3 h in medium containing 1 mCi [γ-^32^P]ATP, and tagged or endogenous G3BP-1 proteins were immunoprecipitated from cell lysates with anti-G3BP sera (Becton-Dickinson Biosciences). Samples were eluted with 25 mm imidazole (pH 7.2), and used for the protein phosphatase activity assay.

### Assay of protein phosphatase activity

HeLa cells were transfected either with expression vectors for native MK-STYX (pMT2-FLAG-MK-STYX-FLAG) or the catalytically active mutant MK-STYX (pMT2-FLAG-MK-STYX_active_-FLAG). Forty-eight hours post-transfection, cells were lysed and MK-STYX proteins were immunoprecipitated using anti-FLAG sera (Sigma). Phosphatase activity in the immunoprecipitates was assayed by incubation with ^32^P-labelled G3BP-1 or G3BP-GFP as the substrate at 30 °C in 25 mm imidazole (pH 7.2), 0.1 mg·mL^−1^ bovine serum albumin and 1 mm dithiothreitol. G3BP-1 was labelled by [γ-^32^P]ATP and immunoprecipitated with anti-G3BP (Becton-Dickinson Biosciences). The reaction was terminated by the addition of a 300 μL charcoal mix (0.9 m NaCl, 90 mm Na_4_P_2_O_7_, 2 mm NaH_2_PO_4_ and charcoal; Sigma) followed by centrifugation and counting of 250 μL supernatant in scintillant. The activity was determined with a LS 6500 Multi-purpose Scintillation Counter (Beckman Coutler, Inc., Fullerton, CA, USA). The presence of immunoprecipitated MK-STYX and the active mutant was confirmed by 10% SDS/PAGE and autoradiography.

### Immunoprecipitation and immunoblotting

Forty-eight hours post-transfection, HeLa cells were harvested in lysis buffer [50 mm HEPES, pH 7.2, 150 mm NaCl, 10% glycerol, 10 mm NaF, 1% Nonidet P-40 alternative (Calbiochem, San Diego, CA, USA) and protease inhibitor cocktail tablets (Roche, Basel, Switzerland)]. Lysates were centrifuged at 14 000 ***g*** for 10 min, and the supernatant protein concentration was determined by NanoDrop quantification (NanoDrop Technologies, Inc., Wilmington, DE, USA). For immunoprecipitation, the lysates were precleared and incubated with anti-FLAG (Sigma) or anti-G3BP (Becton-Dickinson Biosciences) for 1 h at 4 °C, followed by incubation with Protein G beads (GE Healthcare, Little Chalfont, UK). Samples were washed three times in lysis buffer and boiled in Laemmli sample buffer. Lysates were resolved by 10% SDS/PAGE and transferred to a poly(vinylidene difluoride) membrane (GE Healthcare) for immunoblot analysis with anti-FLAG (Sigma) and anti-G3BP sera, followed by chemiluminescent detection. ^32^P-labelled immunoprecipitates were eluted with imidazole and stored for phosphatase analysis. For phospho-G3BP studies, lysates were resolved by 10% SDS/PAGE and transferred to a poly(vinylidene difluoride) membrane with the iBlotter (Invitrogen) for immunoblot analysis with anti-phoshoG3BP (pSer^149^) (Sigma), anti-FLAG (Sigma) and β-tubulin (Pierce, Rockford, IL, USA) sera. Blots were probed first with pSer^149^ (dilution 1 : 1000), and then β-tubulin for the loading control. Aliquots of the same sample lysates were resolved simultaneously and probed for anti-FLAG.

## Abbreviations

DAPI: 4′,6-diamidino-2′-phenylinodole dihydrochloride
FITC: fluorescein isothiocyanate
G3BP-1: Ras-GTPase activating protein SH3 domain binding protein-1
GFP: green fluorescent protein
MAPK: mitogen-activated protein kinase
MBK-2: minibrain kinase homologue-2
MK-STYX: mitogen-activated protein kinase phosphoserine/threonine/tyrosine-binding protein
MTM: myotubularin
NTF2: nuclear transport factor 2
PTP: protein tyrosine phosphatase
